# LIFE-Moms: effects of multicomponent lifestyle randomized control trial on physical activity during pregnancy in women with overweight and obesity

**DOI:** 10.1186/s12966-025-01805-9

**Published:** 2025-09-30

**Authors:** Hannah E. Cabre, Kimberly L. Drews, Jeremy Pomeroy, Sarah Kozey Keadle, S. Sonia Arteaga, Paul W. Franks, Debra Haire-Joshu, William C. Knowler, Xavier Pi-Sunyer, Linda Van Horn, Rena R. Wing, Alison G. Cahill, Rebecca G. Clifton, Kimberly A. Couch, Dympna Gallager, Jami L. Josefson, Kaumudi Joshipura, Samuel Klein, Corby K. Martin, Alan M. Peaceman, Suzanne Phelan, Elizabeth A. Thom, Leanne M. Redman, the LIFE-Moms Research Group, Kimberly L. Drews, Jeremy Pomeroy, S. Sonia Arteaga, Paul W. Franks, Debra Haire-Joshu, William C. Knowler, Xavier Pi-Sunyer, Linda Van Horn, Rena R. Wing, Alison G. Cahill, Rebecca G. Clifton, Kimberly A. Couch, Jami L. Josefson, Kaumudi Joshipura, Samuel Klein, Corby K. Martin, Alan M. Peaceman, Suzanne Phelan, Elizabeth A. Thom, Leanne M. Redman, T. A. Hagobian, A. Schaffner, C. Hart, E. K. Yin, M. G. Phipps, B. Abrams, T. O. Scholl, D. A. Savitz, K. Munoz-Christian, E. Jelalian, D. Gallagher, B. Rosenn, C. Paley, S. Gidwani, M. Horowitz, J. Crane, S. Lin, J. Thornton, M. Holowaty, I. Janumala, J. Johnson, T. Toro-Ramos, E. Widen, W. Yu, M. Campos, M. A. Trak-Fellermeier, M. Meléndez, C. Palacios, J. Pomeroy, I. Febo, J. Vergara, J. Rivera, K. Méndez, R. Torres, S. Soltero, L. Ramos, V. Rivera, W. C. Willett, M. W. Gillman, R. Stein, A. Mathur, W. T. Cade, M. Kwasny, L. Neff, N. Gernhofer, E. Vincent, V. Vignolles, B. Spring, K. Elkind-Hirsh, J. Breaux, D. S. Hsia, J. H. Burton, L. E. Cain, A. A. Altazan, E. F. Sutton, L. A. Gilmore, J. M. Curtis, D. L. Dunnigan, B. A. Grice, R. L. Hanson, M. A. Hoskin, K. G. Kavena, C. Moffett, S. Murphy, J. A. Nelson, R. G. Nelson, S. Sangster, J. R. M. Liao, L. A. Shovestull, S. K. Tanamas, R. Williams, T. Boekhoudt, M. Evans, S. Z. Yanovski, D. L. Alekel, M. Miodovnik

**Affiliations:** 1 Reproductive Endocrinology and Women’s Health Research Program, Pennington Biomedical Research Center, 6400 Perkins Rd, Baton Rouge, LA 70808 USA; 2 The Biostatistics Center, George Washington University, Washington, DC USA; 3 Marshfield Clinic Research Institute, Marshfield, WI USA; 4 Department of Kinesiology & Public Health, California Polytechnic State University, San Luis Obispo, CA USA; 5 ECHO, Office of the Director, National Institutes of Health, Bethesda, MD USA; 6 Department of Nutrition, Harvard T.H. Chan Public Health School, Harvard University, Boston, MA USA; 7 Department of Clinical Sciences, Genetic and Molecular Epidemiology Unit, Lund University Diabetes Centre, Skåne University Hospital Malmö, Malmö, Sweden; 8 Center for Diabetes Translation Research, Washington University in St. Louis, St. Louis, MO USA; 9 Diabetes Epidemiology and Clinical Research Section, National Institute of Diabetes and Digestive and Kidney Diseases, Phoenix, AZ USA; 10 Department of Medicine, New York Obesity Research Center, College of Physicians and Surgeons,, Columbia University, New York, NY USA; 11 Institute of Human Nutrition, College of Physicians and Surgeons, Columbia University, New York, NY USA; 12 Department of Preventive Medicine, Northwestern University, Feinberg School of Medicine, Chicago, IL USA; 13 The Miriam Hospital and the Department of Psychiatry and Human Behavior, Warren Alpert Medical School at Brown University, Providence, RI USA; 14 Department of Women’s Health, University of Texas, Austin, TX USA; 15 Frontier Nursing University, Phoenix, AZ USA; 16 Department of Pediatrics, Northwestern University, Feinberg School of Medicine, Chicago, IL USA; 17 Bagchi School of Public Health, Ahmedabad University, Ahmedabad, India; 18 Department of Epidemiology, Harvard T.H. Chan School of Public Health, Harvard University, Boston, MA USA; 19 Center for Human Nutrition, Washington University in St. Louis, St. Louis, MO USA; 20 Department of Obstetrics and Gynecology, Feinberg School of Medicine, Northwestern University, Chicago, IL USA

**Keywords:** Gestational weight gain, Moderate to vigorous physical activity, Maternal health outcomes, Neonatal health outcomes, Clinical trials

## Abstract

**Background:**

This report details the effect of LIFE-Mom’s multicomponent lifestyle interventions on physical activity (PA) and inactivity time across pregnancy (2nd and 3rd trimesters) and their effect on gestational weight gain (GWG) and maternal/neonatal outcomes, a pre-specified secondary analysis.

**Methods:**

Pregnant people with BMI ≥ 25 kg/m^2^ were randomized to lifestyle interventions with dietary and PA counseling or standard care. PA and inactivity time measured by accelerometry and metabolic and inflammatory biomarkers measured in fasting blood are reported in 522 pregnant people at baseline and end of pregnancy. Generalized linear models with and without covariates were used to evaluate group differences (intervention vs. control) and, separately, time differences (total sample with both groups combined).

**Results:**

Although there were statistically significant differences in vigorous activity between the intervention and control group (*p* = .024), there were no clinically meaningful differences in PA. In the combined sample, moderate to vigorous PA (MVPA) significantly decreased across pregnancy (mean ± SD: 72.9 ± 29.1 min/day vs 63.9 ± 28.1 min/day; *p* < 0.0001), and inactivity time increased [617.5 min/day (573.5, 659.6) vs 630.4 min/day (56.7, 679.9); *p* < 0.0001]. Increased inactivity time was associated with a less favorable maternal milieu (biomarker Z-scores) for pro-inflammatory (0.2 ± 0.1; *p* = 0.003) and cardiometabolic markers (0.1 ± 0.07; *p* = 0.030).

**Conclusions:**

Physical activity declined over the course of pregnancy, though the intervention group experienced a smaller reduction in activity levels. Our results linked increased inactivity time to maternal metabolic dysregulation and inflammation. Further research is needed to determine if intensive interventions reducing inactivity can improve maternal health and weight outcomes in pregnant people with overweight and obesity.

**Trial registration:**

NCT01545934, NCT01616147, NCT01771133, NCT01631747, NCT01768793, NCT01610752, and NCT01812694.

**Supplementary Information:**

The online version contains supplementary material available at 10.1186/s12966-025-01805-9.

## Background

At least two of three women of reproductive age in the US enter pregnancy having overweight or obesity [1]. Pre-pregnancy overweight/obesity coupled with excess weight gain in pregnancy have increased long-term metabolic [2] and cardiovascular disease risks [3], affecting both the mother and child [4]. To address these impacts, the US Preventive Service Task Force issued a grade B recommendation for using multicomponent behavioral interventions (e.g., diet and exercise) in pregnancy to limit excess weight gain [5].

The national physical activity guidelines recommend pregnant people complete at least 150 min (e.g., 30 min a day, five days a week) of moderate-intensity aerobic (i.e., brisk walking) activity per week [6]. Prior trials have tested the benefits of moderate to vigorous physical activity (MVPA) interventions ranging from 8 to 30 weeks and with varying degrees of intensity from supervised, in-person sessions (i.e., high intensity) to encouraging 10,000 steps per day (i.e., low intensity) with self-monitoring [5, 7]. These trials have suggested that physical activity in the perinatal period provides many maternal health benefits, including improved glucose control, reduced systemic inflammation and reduced risks for preeclampsia, gestational hypertension, and excess gestational weight gain [7–9].

The Lifestyle Interventions for Expectant Moms (LIFE-Moms) consortium was established as seven independent but collaborative randomized clinical trials in the United States, collectively enrolling 1,150 pregnant people with overweight or obesity [10]. The goal of the multicomponent interventions which incorporated diet and physical activity modification, and behavior change therapy was to limit weight gain compared with standard care in pregnant people with overweight or obesity. As previously reported in an individual-level meta-analysis, the multicomponent behavioral interventions resulted in a statistically significantly lower incidence of excess gestational weight gain (GWG) compared to standard care [10]. The effect was consistent across the varied lifestyle interventions in a large ethnically and socioeconomically diverse group of pregnant people. A pre-specified secondary analysis was to evaluate intervention impacts on physical activity and inactivity levels and subsequent maternal and neonatal health outcomes.

The primary objective of this analysis was to examine the impact of multicomponent behavioral lifestyle interventions initiated prior to 16 weeks’ gestation in women with overweight or obesity, compared to standard care, on physical activity and inactivity levels measured by accelerometry. Additionally, in the total sample, we examined if physical activity and inactivity levels across pregnancy influenced GWG and pregnancy and neonatal outcomes, specifically in those who either maintained or increased physical activity compared to those who increased inactivity time.

## Methods

### Study design

The LIFE-Moms consortium included seven independent randomized clinical trials. A detailed report of the trials and the consortium outcomes has been previously described [11]. Eligibility confirmation, study visits, procedures, and outcome measures were standardized across all seven clinical trials to allow for data pooling and planned data analyses. Each LIFE-Moms trial consisted of a multicomponent behavioral intervention with physical activity as one of the components.

Participants were enrolled at ≤ 15 weeks, 6 days of gestation (baseline) and subsequently randomized within their respective trial to either a behavioral intervention group (weight, diet, and physical activity counseling) or a comparison group that received standard obstetric care. Physical activity was assessed at baseline and end of pregnancy (3rd trimester; 35 to 36 weeks gestation). At these visits, participants completed demographics & social history questionnaires, had assessments for anthropometrics, blood pressure, fasting biochemistry assays, and habitual physical activity with accelerometry over 7–10 days. At birth, chart abstractions were performed to obtain pregnancy and delivery outcomes, and infant anthropometry was measured (± 14 days after birth). Institutional review boards for each site and the LIFE-Moms Data Safety Monitoring Board approved and monitored the studies. All study participants provided written and informed consent prior to participation.

### Study participants

To be eligible, participants were pregnant with a singleton pregnancy, BMI ≥ 25 kg/m^2^ from study measured weight and height and with a gestational age ≥ 9 weeks, 0 days to ≤ 15 weeks, 6 days at enrollment. Detailed exclusion criteria have been previously reported [11]. For this analysis, participants were also required to have at least 3 days of accelerometry data (> 8 h of wear time) at both the baseline and end of pregnancy time points [12, 13].

### Treatment groups

All interventions included behavioral modification strategies encouraging improvements in diet quality and physical activity and adherence to the 2009 National Academy of Medicine (NAM) weight gain guidelines. A detailed report of the intervention at each site has previously been published [11]. The trials focused on promoting 30 min of physical activity most days of the week (Healthy Beginnings [14], the Lifestyle Intervention for Two [15], the PreGO [16]), encouraging 10,000 steps per day (the Maternal-Offspring Metabolics: Family Intervention Trial [MOMFIT] [17]), provided tools for self-monitoring of physical activity (The Pregnancy and Early Life Improvement Study [PEARLS] [18], Expecting Success [19]) and/or encouraged decreasing sitting time (Phoenix LIFE-Moms). The specific approaches used to support physical activity adoption were self-monitoring, one-on-one counseling, group classes, or e-health counseling and differed among trials [11].

### Procedures

#### Demographic questionnaires

At the baseline visit, participants were asked to complete a demographic and social history questionnaire. Participants self-reported maternal race/ethnicity, current marital status, income status, education status, current living situation, and parity. Other questions included smoking and alcohol use before pregnancy, nausea, and food access.

#### Physical activity measures

Physical activity was assessed at baseline (13,0 to 15,6 weeks gestation) and at the end of pregnancy (35,0 to 36,0 weeks gestation) using the Actigraph GT3X + accelerometer (Actigraph, LLC) worn on the wrist of the non-dominant hand for up to 7 days. Participants in both groups were asked to wear the accelerometer 24 h per day including while they slept and bathed. To accommodate this, participants could request a Velcro strap for the devices for comfort while sleeping. Actigraphs are water-resistant allowing them to remain on during brief water exposure such as showering. All devices were initialized to record at a consistent sampling rate (50 Hz), the highest sampling rate possible for the seven-day monitoring period. The raw files were exported on a minute-by-minute basis with the raw data filtered using the ActiLife proprietary filter. The GGIR application (version 1.11) [13] was used to process the physical activity variables for days when the number of wear hours exceeded 8. The Euclidian Norm Minus One (ENMO) value calculated by GGIR as a proxy for energy expenditure was used to classify level of activity (inactive [< 50.0 mg], light [50.0 mg to ≤ ENMO < 99.9 mg], moderate [100.0 mg to ≤ ENMO < 399.9 mg], vigorous [> 400.0 mg]) and the time spent in each activity [12, 20]. Variables were summarized for each participant and each occasion of wear as counts or non-weighted averages. Data collected from the accelerometer include inactive time (minutes), light activity time (minutes), moderate activity time (minutes), vigorous activity time (minutes), MVPA time (minutes), time spent in MVPA bouts ≥ 1 min, time spent in MVPA bouts ≥ 10 min, number of MVPA bouts ≥ 1 min, awake ENMO (excluding sleep time), and total ENMO [13, 21, 22].

#### Gestational weight gain

Weight was measured at the clinical outcome visits by study staff. Participants were asked to wear a hospital gown, or shoes the morning after an overnight fast. Weight gain (kg) per week was the difference between the weight at baseline and end of pregnancy visit divided by the number of weeks between visits. Second trimester weight gain was the difference between baseline weight and the measured clinic weight at the 24–27 weeks gestation, and third trimester weight gain was the difference between the clinic weights at 24–27 weeks gestation and 35–26 weeks gestation with these differences divided by the number of weeks between visits. Women with baseline weights measured at 14,0—14,6 weeks had 0.45 kg (1 pound) subtracted, and women at 15,0–15,6 weeks had 0.91 kg (2 pounds) subtracted for an estimate of their first-trimester baseline weight [11]. Excess weight gain was defined as weight gain per week above guidelines (overweight > 0.32 kg/week; obesity > 0.27 kg/week).

#### Clinical biochemistry

At baseline and the end of pregnancy, a 30 mL fasting blood sample was collected and analyzed centrally at the Core Laboratory for Clinical Studies (Washington University School of Medicine, St. Louis, MO). Total cholesterol, triglycerides, high-density lipoprotein (HDL), and low-density lipoprotein (LDL) (Roche Cobas C501), blood glucose (Hexokinase UV method), insulin and C-peptide (Roche Cobas E601), leptin (EMD Millipore), adiponectin, interleukin-6 (IL-6), and tumor necrosis factor alpha (TNF-α) (R&D Systems) were measured with standard assays and LDL calculated via the Friedewald equation. Leptin was measured via RIA (EMD Millipore), and adiponectin, IL-6, and TNF-α were measured by ELISA (R&D Systems). Insulin resistance was estimated using the HOMA-IR formula: [fasting insulin (μU/mL) x fasting glucose (mg/dL)/405 [23]. A composite biomarker Z-score for each participant was calculated by standardizing individual biomarker values and summing them, modifying for favorable biomarkers like HDL.

#### Neonatal outcomes

Birth weight was abstracted from the medical records. The newborn was examined by clinic staff within 14 days of life and infant weight, length, birth weight for length z-score calculated using the WHO Child Growth Standards, head circumference, and skinfold thickness at 4 sites: triceps, subscapular, iliac crest, and thigh were measured. Percent body fat was estimated using the Goran equation [24]. Newborns were classified as small for gestational age (SGA) or large for gestational age (LGA), if < 10th percentiles and > 90th percentiles, respectively [25]. Macrosomia was determined if newborns were > 4000 g at birth.

#### Statistical analysis

Of the 1,150 participants enrolled in LIFE-Moms, 628 participants were excluded from this analysis due to skipped measurement (*N* = 508), or fewer than 3 days of accelerometer data having at least 8 h of wear time (*N* = 120) (Supplementary Table 1). Analyses included all other participants regardless of their compliance with the intervention. Physical activity variables were summarized for each participant and each occasion of wear. A threshold of 100.0 mg ENMO was used to define MVPA, where at least 80% of the bouts had to exceed the threshold. Epoch lengths used were five seconds, and bout lengths were 1, 5, and 10 min. Inactive time was defined as time where 0.0 mg ≤ ENMO < 50.0 mg. Descriptive statistics are reported as percent for categorical variables, mean ± standard deviation (SD) for normally distributed continuous variables, and median (Q1, Q3) for skewed continuous variables. For modeling, skewed variables were log or square root transformed to achieve approximate distributional normality. Generalized linear mixed models (GLMM) examining baseline characteristics between participants with overweight or obesity (Table 1) and participants included or excluded in this analysis (Supplementary Table 1) were conducted using a random effect for site unless otherwise noted.

**Table 1:** Baseline characteristics for overall sample and by BMI category

	**Overall** (*N* = 522)	**Overweight** (*N* = 241)	**Obesity** (*N* = 281)	***p*-value**
Maternal age (years)^a^	30.4 ± 5.7	30.7 ± 5.7	30.2 ± 5.7	0.198
Gestational age (weeks)^a^	13.8 ± 1.6	14.1 ± 1.5	13.7 ± 1.7	0.060
Race/Ethnicity^b^				0.028
*Hispanic*	20.5%	20.7%	20.3%	
*Non-Hispanic African American*	37.0%	30.7%	42.3%	
*Non-Hispanic White*	34.7%	38.6%	31.3%	
*Multiracial/Other*	7.9%	10.0%	6.0%	
Income level				0.330
< *$25,000*	37.1%	31.1%	42.2%	
*$25,000 – $74,999*	27.4%	25.7%	28.9%	
≥ *$75,000*	35.5%	43.2%	28.9%	
College degree	47.9%	56.8%	40.2%	0.082
Married/Living with significant other	73.8%	75.5%	72.2%	0.459
Nulliparous	41.0%	49.0%	34.2%	0.005
Randomization assignment				0.031
Control	48.3%	43.2%	52.7%	
Intervention	51.7%	56.8%	47.3%	

Data are presented as Mean ± Standard Deviation or %. Analysis conducted using a random effect for site unless otherwise noted

^a^Adjusted Means ± Standard Error for continuous comparisons – Maternal age: Overweight 30.0 ± 1.2 years, Obese 30.6 ± 1.2 years; Gestational age: Overweight 13.7 ± 0.3 weeks, Obese 13.5 ± 0.3 weeks

^b^No random effect for site included due to lack of race/ethnicity variability within certain sites

To evaluate the impact of the lifestyle intervention compared to standard care on physical activity and inactivity time, outcomes were assessed at the end of pregnancy and changes from baseline using GLMM. These models were adjusted for baseline values, with random effects for study and further adjustments for maternal age, race/ethnicity, parity, and baseline BMI (unadjusted and adjusted *p*-values in Table 2 and Supplementary Table 2). The groups were then combined to assess primary outcomes in the total sample, with similar adjustments and random effects for site.

**Table 2:** Physical activity levels at the end of pregnancy by randomization assignment and characteristics of physical activity at baseline and end of pregnancy for the combined sample

**Randomization Assignment**				
	**Standard of Care (*N* = 252)**	**Intervention (*N* = 270)**	Unadjusted *p*-value*	Adjusted *p*-value**
Inactive time (min/day)^a^	628.5 (588.4, 681.2)	627.9 (589.8, 672.9)	0.353	0.363
Light activity (min/day)	160.8 ± 45.5	168.1 ± 46.1	0.066	0.080
Moderate activity (min/day)	62.4 ± 26.6	65.3 ± 27.8	0.241	0.253
Vigorous activity (min/day)^a^	0.8 (0.34, 1.5)	1.0 (0.5, 2.0)	0.021	0.024
MVPA (min/day)	63.6 ± 27.3	67.0 ± 28.8	0.191	0.244
MVPA in bouts ≥ 1 min (min/day)^b^	10.1 (4.5, 17.1)	11.9 (5.9, 19.2)	0.132	0.154
Awake ENMO (mg/day)	31.2 ± 7.8	32.6 ± 7.9	0.088	0.115
**Combined Sample**				
	**Baseline (*N* = 522)**	**End of Pregnancy (*N* = 522)**	**Unadjusted *p*-value**^**†**^	**Adjusted *p*-value**^**††**^
Inactive time (min/day)^a^	614.3 (570.7, 656.2)	627.9 (588.7, 676.4)	< 0.001	< 0.001
Light activity (min/day)	162.1 ± 45.7	164.6 ± 45.9	0.368	0.353
Moderate activity (min/day)	71.1 ± 28.1	63.9 ± 27.2	< 0.001	< 0.001
Vigorous activity (min/day)^a^	1.2 (0.6, 2.4)	0.9 (0.4, 1.8)	< 0.001	< 0.001
MVPA (min/day)	72.9 ± 29.1	65.4 ± 28.1	< 0.001	< 0.001
MVPA in bouts ≥ 1-min (min/day)^b^	14.8 (8.3, 22.5)	11.0 (5.1, 18.2)	< 0.001	< 0.001
Awake ENMO (mg/day)	33.8 ± 8.2	31.9 ± 7.9	< 0.001	< 0.001

Values presented as mean ± standard deviation or median ± (Q1, Q3). All models adjusted for the baseline value of the activity variable and included a random effect for site (protocol)

^*^Analyses adjusted for baseline value of the activity outcome variable

^**^Analysis adjusted for maternal age, race/ethnicity, parity, baseline BMI category, and baseline value of the activity outcome variable

^†^Analyses adjusted for intervention group

^††^Analysis adjusted for maternal age, race/ethnicity, parity, baseline BMI category, and intervention group

^a^Log transform of the outcome to obtain approximate normality was used for modeling

^b^Square root transform of the outcome to obtain approximate normality was used for modeling

To assess activity exposure during pregnancy, average MVPA time, MVPA time in bouts ≥ 1 min, and inactivity time were calculated at baseline and 35 weeks. The sample was divided into two tertiles (low and high exposure) for MVPA, MVPA bouts, and inactivity time (Table 3; Supplementary Fig. 1, Supplementary Table 3). The sample was also stratified into tertiles to examine changes in MVPA, MVPA bouts, inactivity time, and awake ENMO across gestation. Tertile 1 represented the lowest activity or highest inactivity, while Tertile 3 represented the highest activity or lowest inactivity. Percentages within each tertile assessed increases or decreases in MVPA and inactivity time. Cut-off points for the tertiles of each outcome variable are located on Table 3 and in the footnote for Supplementary Table 4.

**Table 3:** Relationship of gestational weight gain with pregnancy activity levels

	**Tertile 1 (*N* = 173)**	**Tertile 3 (*N* = 171)**	Unadjusted *p*-value*	Adjusted *p*-value**
Total MVPA During Pregnancy				
Gestational Weight Gain (kg)	8.68 ± 5.02	8.84 ± 5.12	0.59	0.76
Gestational Weight Gain/week (kg/wk)	0.39 ± 0.23	0.38 ± 0.22	0.91	0.91
Excess GWG per Week	65.5%	67.9%	0.49	0.67
2nd Trimester GWG per Week (kg/wk)	0.38 ± 0.26	0.36 ± 0.24	0.76	0.89
Excess 2nd Trimester GWG per Week	63.0%	63.9%	0.64	0.71
3rd Trimester GWG per Week (kg/wk)	0.40 ± 0.26	0.42 ± 0.28	0.39	0.62
Excess 3rd Trimester GWG per Week	61.2%	68.2%	0.16	0.46
MVPA in Bouts ≥ 1 Minute During Pregnancy				
Gestational Weight Gain (kg)	8.64 ± 5.37	9.83 ± 4.92	0.01	0.10
Gestational Weight Gain/week (kg/wk)	0.38 ± 0.24	0.43 ± 0.21	0.040	0.13
Excess GWG per Week	65.0%	72.2%	0.08	0.26
2nd Trimester GWG per Week (kg/wk)	0.39 ± 0.28	0.39 ± 0.22	0.66	0.87
Excess 2nd Trimester GWG per Week	64.8%	69.0%	0.50	0.75
3rd Trimester GWG per Week (kg/wk)	0.39 ± 0.28	0.47 ± 0.28	< 0.01	0.07
Excess 3rd Trimester GWG per Week	59.4%	74.2%	< 0.01	0.02
Inactive Time During Pregnancy				
Gestational Weight Gain (kg)	8.73 ± 5.09	9.24 ± 5.16	0.52	0.83
Gestational Weight Gain/week (kg/wk)	0.38 ± 0.22	0.42 ± 0.23	0.24	0.81
Excess GWG per Week	66.1%	69.7%	0.62	0.88
2nd Trimester GWG per Week (kg/wk)	0.35 ± 0.24	0.44 ± 0.26	0.01	0.12
Excess 2nd Trimester GWG per Week	62.5%	73.1%	0.12	0.45
3rd Trimester GWG per Week (kg/wk)	0.41 ± 0.26	0.41 ± 0.27	0.93	0.36
Excess 3rd Trimester GWG per Week	66.9%	67.6%	0.98	0.93

Variables were calculated as [(baseline + end of prengancy)/2] and presented as the bottom and top tertiles. Summary variables are presented as mean± SD or %

Total MVPA During Pregnancy Range: Tertile 1=18.20–55.80 min/day and Tertile 3= 80.40–171.25 min/day

MVPA in Bouts ≥ 1 Minute During Pregnancy Range: Tertile 1=0.60–9.30 min/day and Tertile 3=18.00-44.45 min

Inactive Time During Pregnancy Range: Tertile 1= 443.10-598.25 min and Tertile 3=649.60-851.20 min

^*^Models only adjusted for treatment assignment

^**^Models adjusted for treatment assignment, maternal age, race/ethnicity, parity, baseline BMI category

To evaluate GWG in the combined sample, GLMM were used, including random effects for site and adjustments for intervention group and baseline values (unadjusted and adjusted *p*-values in Table 4). These models assessed the effects of changes in physical activity and inactivity time from baseline to the end of pregnancy on GWG, GWG per week, and second- and third-trimester GWG per week. Odds ratios were used for associations with dichotomous variables (e.g., excess GWG per week), while slopes and standard errors described relationships with continuous variables (e.g., total GWG). Additional models compared activity changes between top and bottom tertiles during pregnancy, adjusted for maternal characteristics (Supplementary Table 3).

**Table 4:** Relationship between change in activity levels (end of pregnancy—baseline) and gestational weight gain (GWG) in the total sample

**Outcomes**	**Change in Inactive Time**				**Change in Total MVPA**			
	**Unadjusted Model**^**a**^		**Adjusted Model**^**b**^		**Unadjusted Model**^**a**^		**Adjusted Model**^**b**^	
	**β ± SE or** **OR (95%CI)**	***p*-value**	**β ± SE or** **OR (95%CI)**	***p*-value**	**β ± SE or** **OR (95%CI)**	***p*-value**	**β ± SE or** **OR (95%CI)**	***p*-value**
GWG (kg)	−0.10 ± 0.09	0.267	−0.07 ± 0.09	0.431	0.08 ± 0.05	0.102	0.05 ± 0.05	0.333
GWG/Week (kg/wk)	−0.07 ± 0.09	0.396	−0.06 ± 0.08	0.514	0.08 ± 0.05	0.108	0.05 ± 0.05	0.295
2nd Trimester GWG/Week (kg/wk)	−0.00 ± 0.10	0.962	−0.00 ± 0.09	0.967	0.06 ± 0.05	0.262	0.04 ± 0.05	0.400
3rd Trimester GWG/Week (kg/wk)	−0.04 ± 0.10	0.655	0.01 ± 0.09	0.934	0.12 ± 0.05	0.024	0.08 ± 0.05	0.149
Excess GWG/Week	0.69 (0.46, 1.03)	0.071	0.68 (0.44, 1.03)	0.070	1.39 (1.10, 1.75)	0.005	1.38 (1.08, 1.76)	0.011
Excess 2nd Trimester GWG/Week	0.85 (0.56, 1.30)	0.456	0.84 (0.54, 1.31)	0.444	1.18 (0.93, 1.50)	0.175	1.20 (0.93, 1.54)	0.160
Excess 3rd Trimester GWG/Week	0.99 (0.65, 1.50)	0.952	1.13 (0.73, 1.46)	0.576	1.14 (0.89, 1.45)	0.299	1.05 (0.81, 1.35)	0.726
	**Change in MVPA Bouts ≥ 1 Minute**				**Change in Awake ENMO**			
	**Unadjusted Model**^**a**^		**Adjusted Model**^**b**^		**Unadjusted Model**^**a**^		**Adjusted Model**^**b**^	
	**β ± SE or** **OR (95%CI)**	***p*-value**	**β ± SE or** **OR (95%CI)**	***p*-value**	**β ± SE or** **OR (95%CI)**	***p*-value**	**β ± SE or** **OR (95%CI)**	***p*-value**
GWG (kg)	0.14 ± 0.05	0.010	0.09 ± 0.05	0.087	0.07 ± 0.05	0.124	0.04 ± 0.05	0.338
GWG/Week (kg/wk)	0.14 ± 0.05	0.010	0.10 ± 0.05	0.065	0.07 ± 0.05	0.133	0.05 ± 0.05	0.325
2nd Trimester GWG/Week (kg/wk)	0.06 ± 0.06	0.279	0.03 ± 0.06	0.582	0.05 ± 0.05	0.308	0.04 ± 0.05	0.477
3rd Trimester GWG/Week (kg/wk)	0.20 ± 0.06	0.001	0.14 ± 0.06	0.016	0.11 ± 0.05	0.040	0.07 ± 0.05	0.160
Excess GWG/Week	1.39 (1.08, 1.79)	0.012	1.34 (1.02, 1.75)	0.035	1.41 (1.13, 1.76)	0.003	1.40 (1.11, 1.77)	0.005
Excess 2nd Trimester GWG/Week	1.06 (0.82, 1.36)	0.681	1.04 (0.79, 1.36)	0.783	1.19 (0.95, 1.50)	0.137	1.20 (0.94, 1.53)	0.148
Excess 3rd Trimester GWG/Week	1.34 (1.02, 1.75)	0.037	1.22 (0.92, 1.62)	0.165	1.10 (0.88, 1.38)	0.413	1.03 (0.81, 1.31)	0.833

Odds ratios are per 1 SD increase in the activity/inactivity variable

^a^Models adjusted for treatment assignment, the baseline value for the independent variable (activity or inactivity measure)

^b^Models adjusted for treatment assignment, the baseline value for the independent variable (activity or inactivity measure), maternal age, race/ethnicity, parity, baseline BMI category

The effects of changes in physical activity and inactivity time during pregnancy on maternal and neonatal outcomes were assessed using GLMM. These models were adjusted for baseline values and intervention assignment (unadjusted *p*-values) and further adjusted for maternal race/ethnicity, parity, and baseline BMI (adjusted *p*-values), with all models including a random effect for site (Supplementary Table 5). For all outcomes, nominal *p*-values of < 0.05 were considered to indicate statistical significance; *p*-values were not adjusted for multiple comparisons. Analyses were performed using SAS version 9.4 (SAS Institute, Cary, North Carolina).

## Results

### Sample characteristics

At baseline (randomization), participants were 30.4 ± 5.7 (mean ± SD) years and 13.8 ± 1.6 weeks gestational age. Compared to pregnant people who had obesity, those with overweight were more likely nulliparous (*p* = 0.005), non-Hispanic Caucasian and multiracial/other race (*p* = 0.028), and in the intervention group (*p* = 0.031) (Table 1). Only 9.4% of the cohort met the Physical Activity Guidelines of at least 30 min of MVPA per week at baseline with less than 50% of participants having bouts of activity that exceeded 10 min at baseline or weeks 35–36.

#### Physical activity levels by randomization assignment (ITT analysis)

A total of 522 participants were included in this analysis with 51.7% in the intervention group and 48.3% in the standard care group (Table 1). Participants in the intervention group had statistically significant but extremely modest greater total time spent in vigorous physical activity at the end of pregnancy compared to the standard care group [median (Q1, Q3): 1.0 min/day (0.5, 2.0) vs. 0.8 min/day (0.4, 1.5); *p* = 0.024]. There were no other differences between randomized groups for any of the measures of intensities of physical activity or inactive time (Table 2; Supplementary Table 2).

#### Physical activity levels for the combined sample

The total sample provided 5,602 valid physical activity days across both visits with 2,817 days available at baseline visit and 2,785 available at the end of pregnancy. Since there were no differences in PA measures between the intervention and standard care groups, we combined the groups for further analysis (Table 2). In the combined sample, total time spent in moderate activity (*p* < 0.001), vigorous activity (*p* < 0.001), total MVPA (*p* < 0.001), MVPA bouts of at least 1 min (*p* < 0.001), awake ENMO (*p* < 0.001), and total ENMO (*p* = 0.013), all decreased from baseline to the end of pregnancy. Conversely, total inactive time (*p* < 0.001) increased from baseline to the end of pregnancy.

#### Physical activity exposure during pregnancy for the combined sample

To assess changes in activity across pregnancy, the sample was divided into three tertiles based on MVPA and inactivity time (Supplementary Fig. 1). As there were no changes in participants’ activity and inactivity, we calculated the activity exposure across pregnancy [(baseline + end of pregnancy)/2]. There was a significant relationship with GWG, with participants who had an increase in total MVPA [OR 2.00 (1.2, 3.40); *p* = 0.010] and awake ENMO [OR 2.1 (1.3, 3.5); *p* < 0.010] activity exposure across pregnancy being more likely to have excess GWG per week than those who did not increase total MVPA or awake ENMO (Supplementary Table 3). When separated into tertiles 1 (low exposure) and 3 (high exposure) based on activity exposure during pregnancy, those who had the greatest exposure in MVPA in bouts ≥ 1 min (tertile 3) had significantly greater excess GWG in the third trimester (+ 1.2 kg; *p* = 0.020) (Table 3).

#### Physical activity and gestational weight gain across pregnancy in the total sample

Participants who increased their MPVA across pregnancy were more likely to have excess total GWG per week [OR 1.38 (1.08, 1.76); *p* = 0.011] compared to those who decreased MVPA across pregnancy. Similarly, compared to those who decreased MVPA bouts ≥ 1 min across pregnancy*,* participants who had higher time spent in MVPA bouts ≥ 1 min across pregnancy were more likely to have a greater excess GWG per week [OR 1.34 (1.02, 1.75); *p* = 0.035] and greater rate of GWG in the third trimester (*β* ± standard error: 0.14 kg/week ± 0.06 kg/week; *p* = 0.016) (Table 4). For each SD increase in awake time ENMO across pregnancy compared to baseline, the odds of having excess GWG increased 1.4 times per week [OR 1.40 (1.10, 1.80); *p* = 0.005]. There were no significant associations between excess GWG and inactivity time (Table 4).

#### Associations of physical activity tertiles and GWG across pregnancy

**Fig. 1:**
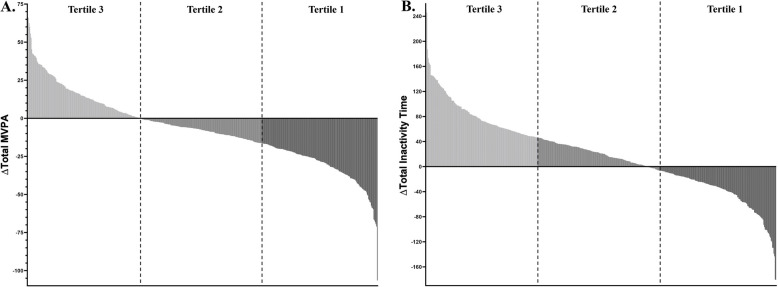
**A** Change in total MVPA minutes (end of pregnancy- baseline) for the total sample. Tertile indicates participants who had the lowest total MVPA at the end of pregnancy (range: −16.4 to −106.5 min; *n* = 171) and Tertile 3 indicates participants who had the highest total MVPA at the end of pregnancy (range: −0.3 to 66.6; *n* = 174). **B** Change in inactivity time (end of pregnancy- baseline) for the total sample. Tertile 1 indicates participants who had the highest total inactivity time at the end of pregnancy (range: 46.8 to 250.7 min; *n* = 166) and Tertile 3 indicates participants who had the lowest inactivity time at the end of pregnancy (range: −6.5 to −180.7 min; *n* = 174)

When separated into tertiles for those who had the highest total MVPA at the end of pregnancy (tertile 1) to those who had the lowest total MVPA at the end of pregnancy (tertile 3), there were no differences in total GWG (*p* = 0.517–0.983), rate of GWG per week (*p* = 0.531–0.949), or excess GWG (*p* = 0.230–0.391) (Fig. 1). Total GWG and the rate of GWG were not different between those who sustained/improved total MVPA and MVPA in bouts ≥ 1 min across pregnancy compared to those who did not improve (*p* = 0.462–0.966) (Supplementary Table 4). Total GWG and the rate of GWG were not different when comparing participants who became more inactive (*p* = 0.056–0.155) across pregnancy compared to those who maintained inactivity time across pregnancy (Supplementary Table 4).

#### Associations of the change in physical activity and GWG across pregnancy

When separated into tertiles for those who had the highest total MVPA at the end of pregnancy (tertile 1) and compared to those who had the lowest total MVPA at the end of pregnancy (tertile 3), there were no differences in total GWG (*p* = 0.057), excess GWG (*p* = 0.059) or the rate of GWG per week (*p* = 0.044) (Supplementary Table 4; Fig. 1). Excess GWG was similar between those who sustained/improved total MVPA (71.3%) and MVPA in bouts ≥ 1 min (71.2%) across pregnancy compared to those who experienced a reduction (65.9% and 66.1%, respectively; *p* = 0.158–0.207) across pregnancy. Excess GWG per week was significantly higher in those who sustained/improved (74.7%) across pregnancy compared to a reduction (63.6%; *p* = 0.011) inactivity time across pregnancy.

#### Analysis of physical activity characteristics associated with maternal and neonatal outcomes

Participants who increased total MVPA during pregnancy had higher total cholesterol (0.07 ± 0.03 mg/dL, *p* = 0.030), whereas those with increased inactivity time had higher maternal metabolic/pro-inflammatory biomarker Z-scores (0.2 ± 0.1, *p* = 0.003). No other significant associations between physical activity and biomarkers were found. Changes in inactivity, total MVPA, MVPA in bouts ≥ 1 min, and awake ENMO did not significantly affect rates of gestational diabetes, hypertension, or preeclampsia (*p* = 0.155–0.665). Infant outcomes, including birth weight and various skinfold measures, were similar regardless of maternal MVPA changes, except for higher MVPA in bouts ≥ 1 min was associated with lower birth weight Z-scores (−0.1 ± 0.1, *p* = 0.032).

## Discussion

Excess weight gain in pregnant people with overweight and obesity increases the risk of adverse maternal and neonatal outcomes [26]. Multicomponent lifestyle interventions with dietary and physical activity modification have shown potential for reducing excess weight gain and promoting healthier pregnancies [27]. In the LIFE-Moms consortium, each of the seven multicomponent behavior-change interventions designed to reduce weight gain included a physical activity component of varying intensity. This secondary analysis revealed that physical activity declined across pregnancy for the intervention and standard care groups similarly. There were two notable findings. First and surprisingly, participants with higher MVPA at the end of pregnancy tended to experience higher gestational weight gain. Second and importantly, more inactivity during pregnancy was associated with less favorable maternal metabolic profiles and increased infant birth weight.

Physical activity during pregnancy has been shown to improve glucose control [28], lower weight gain [29], and reduce the risks for preeclampsia and preterm birth [9]. Thus, the United States Preventive Service Task Force recommended behavioral interventions for preventing excess weight gain [5]. In the current study, the behavioral interventions had limited effects on physical activity, with only minimal changes in vigorous activity, which are not clinically relevant (−0.2 min/day). Despite our large, diverse sample averaging 73 min of MVPA daily at baseline, physical activity dropped by 10% during the 2nd and 3rd trimesters, contributing to over 10 h of inactivity per day. One investigation evaluating behavioral change interventions targeting physical activity and diet in 183 pregnant women with obesity demonstrated similar baseline activity values, but in contrast a 40% decrease in MVPA [30].

A recent systematic review of observational studies across BMI categories of pregnant people found that only 50% of the included studies showed a significant association between various aspects of physical activity and excessive weight gain [31]. However, in LIFE-Moms, physical activity was not associated with a reduction in gestational weight gain; moreover, those who had increased their MVPA by the end of pregnancy were more likely to experience excess weight gain (*p* = 0.008–0.024), although the difference was extremely small (−0.02 kg). Since less than 50% of the total sample lacked sufficient data for 10-min bouts of MVPA, the relationship between physical activity and weight gain could not be fully explored. As such, this analysis most likely reflects lifestyle activity of pregnant people who may not have dedicated exercise time.

Inactivity time significantly increased across pregnancy. Our values of inactivity time align with previous investigations in pregnant people (range: 420–760 min/day) [7, 32, 33]. In a separate secondary analysis of the standard of care group (*n* = 439) from LIFE-moms that examined 24-h movement behaviors (physical activity, sleep, sedentary behavior), MVPA and sleep decreased from early to late pregnancy while sedentary behavior increased [33]. These behavioral patterns demonstrated how participants spent their days and nights. We speculate that behavioral choices from week to week contributed to excess weight gain in the present study despite increases in MVPA but caution the interpretation of these results as the sample of women were largely inactive across pregnancy compared to the recommended MVPA guidelines [34].

Maternal overweight and obesity promote inflammation and higher risk of pregnancy complications like gestational hypertension, preeclampsia, and dyslipidemia [35–38]. Few studies have evaluated the association between a behavioral intervention including physical activity with multiple measures of maternal health [28, 37]. While our study may not have sufficient power to detect changes in these outcomes, inactivity was associated with worse maternal metabolic profiles, emphasizing the importance of maintaining activity during pregnancy. Similar to our findings, one study assessing inactivity time with an ActiGraph accelerometer from 15 weeks to the end of pregnancy in 46 pregnant women with overweight and obesity also demonstrated significant increases in similar biomarkers of fasting insulin, insulin sensitivity, lipid profiles, and cytokines [28].

Though infant birth weight is traditionally used to assess intrauterine health, recent research suggests that body composition might be a better predictor of future health outcomes [39, 40]. We observed no differences in infant birth weight or body composition measures between infants born to mothers who increased or maintained total MVPA compared to those who decreased total MVPA. Other studies have also found no strong link between maternal PA and infant body composition, though some evidence suggests physical activity in late pregnancy may impact infant outcomes [41]. Interestingly, greater MVPA time in bouts ≥ 1 min was associated with lower infant birth weight z-scores, consistent with previous studies.

Dietary data was not collected in all our studies limiting our assessment of potential weight compensation effects. However, three sites conducted 24-h food recalls at baseline and end of pregnancy. In this subsample (*n* = 316/522), there were minimal differences in energy intake across pregnancy for tertile 1 and tertile 3 in MVPA (34.3 ± 639.2 kcal/day and −1.4 ± 722.6 kcal/day, respectively) and inactivity time (62.4 ± 626.8 kcal/day and 0.8 ± 656.5 kcal/day, respectively). Uniquely, the demographic distribution of these data from 316 participants were vastly different than the total sample with 47.8% identifying as Non-Hispanic White (total sample: 34.7%) and only 14.6% identifying as Non-Hispanic Black (total sample: 37.0%). Income distribution was also skewed towards higher socioeconomic status with 49.5% reporting an income greater than or equal to $75,000 (total sample: 35.5%) and only 13.7% reporting an income less than $25,000 (total sample: 37.1%). These data highlight the potential influence of race/ethnicity and socioeconomic status in our results, with previous research citing underserved individuals may have greater barriers to physical activity intervention success such as unsafe neighborhoods limiting the opportunities to exercise, increased occupational time demands, and reduced access to exercise equipment [42].

An important strength of the LIFE-Moms consortium studies was the diversity among the participants in terms of racial groups, and socioeconomical statuses providing excellent generalizability to the US population [10, 11, 38]. The use of objective measure of PA was an additional strength. However, the interventions varied in intensity and delivery (e.g., self-monitored PA, in-person counseling). Additionally, participant adherence with the PA assessment was not uniform across centers (628 participants did not have sufficient data for inclusion in the present analysis) [10]. Records of physical activity measured through delivery ranged from 18.3% compliance to 80.0% depending on the site. Given the inconsistency with the dietary assessment methodology our capacity to draw definitive conclusions about the relationship between physical activity and dietary compensation is hampered. Additionally, the timing of the interventions may have been too late in pregnancy to affect outcomes [43].

Although the LIFE-Moms multicomponent lifestyle interventions incorporated physical activity guidance, there were not meaningful increases in activity throughout pregnancy, and in some instances, greater MVPA was unexpectedly linked to higher gestational weight gain. Future research should explore more structured interventions and focus on decreasing inactivity time, to better align with the physiological and behavioral changes that influence weight gain and activity patterns throughout gestation. Earlier interventions may also offer a critical window to modify behaviors before excessive gestational weight gain begins, and to support long-term health trajectories for both mother and infant [44]. Interventions should be culturally relevant and with an appropriate level of health literacy for increased access and adoption. Ultimately, a shift toward earlier, multi-dimensional, and individually tailored interventions may offer a more promising pathway for improving maternal and neonatal outcomes in populations at high risk.

## Conclusions

In summary, this pre-specified secondary analysis of seven lifestyle interventions suggests the multicomponent behavioral lifestyle interventions that included some guidance for physical activity modification did not meaningfully impact physical activity outcomes in pregnant people with overweight and obesity who may have been inactive prior to beginning the intervention. While LIFE-Moms found that the lifestyle interventions were effective at reducing rates of gestational weight gain [10], our results demonstrated that the physical activity interventions among less low active pregnant people with overweight and obesity did not mitigate weight gain or promote guideline attainment. Furthermore, there were no associations between changes in physical activity and maternal or neonatal outcomes overall. Our results showed associations between inactivity time and the maternal milieu, particularly related to metabolic dysregulation and inflammation. Thus, more studies are needed to evaluate whether an intensive, structured intervention approach targeting reduced inactivity time may produce greater benefit for the maternal milieu, in addition to weight gain, in pregnant people living with overweight and obesity.

## Supplementary Information

Supplementary Material 1.

Supplementary Material 2.

Supplementary Material 3.

Supplementary Material 4.

Supplementary Material 5.

Supplementary Material 6.

## Data Availability

Data Availability: Lifestyle Interventions For Expectant Mothers (LIFE-Moms) was conducted by the LIFE-Moms Research Group and supported by the National Institute of Diabetes and Digestive and Kidney Diseases (NIDDK), the National Heart, Lung, and Blood Institute (NHLBI), the Eunice Kennedy Shriver National Institute of Child Health and Human Development (NICHD), the National Center for Complementary and Integrative Health (NCCIH), the NIH Office of Research in Women’s Health (ORWH), the Office of Behavioral and Social Science Research (OBSSR), the NIH Office of Disease Prevention (ODP), the Indian Health Service, the Intramural Research Program of the NIDDK, and the Office of the Director, National Institutes of Health (OD). The data from LIFE-Moms are available at the NIDDK Central Repository (NIDDK-CR), Resources for Research (R4R) at https://repository.niddk.nih.gov/studies/life-moms/.

## Abbreviations

MVPA: Moderate to vigorous physical activity
LIFE-Moms: The Lifestyle Interventions for Expectant Moms
GWG: Gestational weight gain
ENMO: Euclidian Norm Minus One
GLMM: Generalized linear mixed models
