# Supplementary material for: LIFE-Moms: effects of multicomponent lifestyle randomized control trial on physical activity during pregnancy in women with overweight and obesity
Source: Int J Behav Nutr Phys Act. 2025 Sep 30;22:119. doi: 10.1186/s12966-025-01805-9 (PMC12486678; doi:10.1186/s12966-025-01805-9)
Supplement: Supplementary file 1 — Supplementary Material 1. [file 12966_2025_1805_MOESM1_ESM.docx]

| **Supplement Table 1.** Baseline characteristics for overall and by included and excluded from analysis presented as mean ± standard deviation or %. | | | | | | | | | |
| --- | --- | --- | --- | --- | --- | --- | --- | --- | --- |
|  | **Overall** (N= 1,150) | |  | **Excluded** (N=628) | | **Included** (N=522) | |  | **p-value** |
| Maternal age (years)* | 30.4 | ± 5.7 |  | 30.4 | ± 5.7 | 30.4 | ± 5.7 |  | **0.032** |
| Gestational age (weeks)* | 13.8 | ± 1.7 |  | 13.7 | ± 1.8 | 13.8 | ± 1.6 |  | 0.250 |
| Maternal weight (kg)* | 84.9 | ± 15.0 |  | 85.5 | ± 15.3 | 84.2 | ± 14.7 |  | 0.119 |
| BMI (kg/m^2^)* | 31.8 | ± 4.9 |  | 31.9 | ± 4.9 | 31.6 | ± 4.9 |  | 0.160 |
| BMI Category |  | |  |  | |  | |  | 0.106 |
| Overweight | 43.9% | |  | 42.0% | | 46.2% | |  |  |
| Obese | 56.1% | |  | 58.0% | | 53.8% | |  |  |
| Race/Ethnicity** |  | |  |  | |  | |  | **0.010** |
| Hispanic | 23.5% | |  | 26.0% | | 20.5% | |  |  |
| Non-Hispanic African American | 32.4% | |  | 28.7% | | 37.0% | |  |  |
| Non-Hispanic Caucasian | 35.0% | |  | 35.2% | | 34.7% | |  |  |
| Multiracial/Other | 9.1% | |  | 10.2% | | 7.9% | |  |  |
| Income level |  | |  |  | |  | |  | 0.248 |
| < $25,000 | 35.7% | |  | 34.6% | | 37.1% | |  |  |
| $25,000 – $74,999 | 27.2% | |  | 27.0% | | 27.4% | |  |  |
| $75,000+ | 37.1% | |  | 38.4% | | 35.5% | |  |  |
| College degree | 49.7% | |  | 51.1% | | 47.9% | |  | 0.386 |
| Married/Living with significant other | 76.2% | |  | 78.1% | | 73.8% | |  | 0.787 |
| Nulliparous | 41.1% | |  | 41.2% | | 41.0% | |  | 0.657 |
| Randomization assignment |  | |  |  | |  | |  | 0.395 |
| Control | 49.7% | |  | 50.8% | | 48.3% | |  |  |
| Intervention | 50.3% | |  | 49.2% | | 51.7% | |  |  |
| Gestational diabetes | 11.5% | |  | 11.1% | | 12.0% | |  | 0.744 |
| Gestational hypertension/preeclampsia | 14.5% | |  | 16.7% | | 11.9% | |  | **0.004** |
| Preeclampsia | 7.7% | |  | 10.4% | | 4.6% | |  | <**0.001** |
| Shoulder dystocia | 1.0% | |  | 0.8% | | 1.2% | |  | 0.562 |
| Birth trauma | 0.4% | |  | 0.2% | | 0.6% | |  | 0.278 |
| Analysis conducted using a random effect for site unless otherwise noted.  * Adjusted Means ± SE for continuous comparisons – Maternal age: Excluded 29.5 ± 1.2 years, included 30.1 ± 1.2 years; Gestational age: Excluded 13.5 ± 0.3 weeks, included 13.7 ∓ 0.3 weeks; Maternal Weight: Excluded 85.9 ± 1.0 kg, included 84.5 ± 1.1 kg; BMI: Excluded 32.3 ± 0.6 kg/m2, Included 31.9 ± 0.6 kg/m2  ** No random effect for site included due to lack of race/ethnicity variability within certain sites | | | | | | | | | |
