# Supplementary material for: LIFE-Moms: effects of multicomponent lifestyle randomized control trial on physical activity during pregnancy in women with overweight and obesity
Source: Int J Behav Nutr Phys Act. 2025 Sep 30;22:119. doi: 10.1186/s12966-025-01805-9 (PMC12486678; doi:10.1186/s12966-025-01805-9)
Supplement: Supplementary file 2 — Supplementary Material 2. [file 12966_2025_1805_MOESM2_ESM.docx]

| **Supplementary Table 2.** Difference in activity (end of pregnancy – baseline) by treatment assignment. | | | | | |
| --- | --- | --- | --- | --- | --- |
|  | **Control** (N=252) | **Intervention** (N=270) |  | **Unadjusted p-value*** | **Adjusted p-value**** |
| Awake ENMO (mg) | -2.2 ± 6.6 | -1.6 ± 6.8 |  | 0.088 | 0.109 |
| Asleep ENMO (mg) # | 0.4 ± 2.8 | -0.1 ± 2.7 |  | 0.071 | 0.094 |
| Inactive time (min) # | 21.7 ± 64.0 | 18.6 ± 56.7 |  | 0.330 | 0.348 |
| Time spent in light activity (min) | -0.08 ± 41.4 | 4.9 ± 42.5 |  | 0.066 | 0.080 |
| Time spent in moderate activity (min) | -8.2 ± 21.4 | -6.4 ± 23.2 |  | 0.241 | 0.253 |
| Time spent in vigorous activity (min)# | -0.4 ± 1.6 | -0.2 ± 2.0 |  | **0.011** | **0.015** |
| Time spent in MVPA (min) | -8.5 ± 22.0 | -6.6 ± 23.9 |  | 0.191 | 0.206 |
| Time spent in MVPA bouts ≥ 1 min ## | -3.9 ± 7.3 | -3.3 ± 8.3 |  | 0.075 | 0.081 |
| Number of MVPA bouts ≥ 1 min## | -2.0 ± 4.1 | -1.6 ± 4.6 |  | 0.130 | 0.139 |
| Values presented as mean ± SD. All models adjusted for the baseline value of the activity variable and included a random effect for site (protocol).  * Analysis adjusted for maternal age, race/ethnicity, parity, baseline BMI category, and baseline value of the activity outcome variable | | | | | |
