# Supplementary material for: LIFE-Moms: effects of multicomponent lifestyle randomized control trial on physical activity during pregnancy in women with overweight and obesity
Source: Int J Behav Nutr Phys Act. 2025 Sep 30;22:119. doi: 10.1186/s12966-025-01805-9 (PMC12486678; doi:10.1186/s12966-025-01805-9)
Supplement: Supplementary file 3 — Supplementary Material 3. [file 12966_2025_1805_MOESM3_ESM.docx]

| **Supplementary Table 3**. Relationship between activity levels throughout pregnancy [(baseline + end of pregnancy)/2] and gestational weight gain (GWG). | | | | | | | | |
| --- | --- | --- | --- | --- | --- | --- | --- | --- |
|  | **Inactive Time During Pregnancy** | | | | **Total MVPA During Pregnancy** | | | |
|  | Unadjusted Model* | | Adjusted Model** | | Unadjusted Model* | | Adjusted Model** | |
|  | β ± SE or  OR (95%CI) | p-value | β ± SE or  OR (95%CI) | p-value | β ± SE or  OR (95%CI) | p-value | β ± SE or  OR (95%CI) | p-value |
| GWG | -0.10 ± 0.10 | 0.33 | -0.07 ± 0.10 | 0.46 | 0.18 ± 0.11 | 0.10 | 0.10 ± 0.10 | 0.33 |
| GWG/Week | -0.07 ± 0.10 | 0.51 | -0.05 ± 0.09 | 0.57 | 0.17 ± 0.11 | 0.11 | 0.11 ± 0.10 | 0.30 |
| Excess GWG/Week | 0.67 (0.43, 1.05) | 0.08 | 0.65 (0.41, 1.04) | 0.07 | **2.04 (1.24, 3.38)** | **<0.01** | **2.00 (1.18, 3.40)** | **0.01** |
| 2^nd^ Trimester GWG/Week | 0.03 ± 0.11 | 0.76 | 0.02 ± 0.10 | 0.84 | 0.13 ± 0.12 | 0.26 | 0.10 ± 0.11 | 0.40 |
| Excess 2^nd^ Trimester GWG/Week | 0.88 (0.55, 1.40) | 0.58 | 0.86 (0.52, 1.40) | 0.53 | 1.43 (0.85, 2.39) | 0.18 | 1.48 (0.86, 2.55) | 0.16 |
| 3^rd^ Trimester GWG/Week | -0.05 ± 0.11 | 0.67 | 0.01 ± 0.10 | 0.94 | **0.27 ± 0.12** | **0.02** | 0.17 ± 0.12 | 0.15 |
| Excess 3^rd^ Trimester GWG/Week | 0.98 (0.62, 1.55) | 0.92 | 1.13 (0.70, 1.84) | 0.62 | 1.32 (0.78, 2.22) | 0.30 | 1.10 (0.64, 1.91) | 0.73 |
|  | **MVPA Bouts ≥ 1 Minute During Pregnancy** | | | | **Awake ENMO During Pregnancy** | | | |
|  | Unadjusted Model* | | Adjusted Model** | | Unadjusted Model* | | Adjusted Model** | |
|  | β ± SE or  OR (95%CI) | p-value | β ± SE or  OR (95%CI) | p-value | β ± SE or  OR (95%CI) | p-value | β ± SE or  OR (95%CI) | p-value |
| GWG | 0.18 ± 0.11 | 0.09 | 0.11 ± 0.10 | 0.28 | 0.16 ± 0.10 | 0.12 | 0.10 ± 0.10 | 0.34 |
| GWG/Week | 0.18 ± 0.10 | 0.08 | 0.13 ± 0.10 | 0.20 | 0.16 ± 0.10 | 0.13 | 0.10 ± 0.10 | 0.32 |
| Excess GWG/Week | **1.65 (1.02, 2.68)** | **0.04** | 1.57 (0.95, 2.60) | 0.08 | **2.09 (1.29, 3.38)** | **<0.01** | **2.08 (1.25, 3.46)** | **<0.01** |
| 2^nd^ Trimester GWG/Week | 0.06 ± 0.11 | 0.59 | 0.04 ± 0.11 | 0.73 | 0.12 ± 0.11 | 0.31 | 0.08 ± 0.11 | 0.48 |
| Excess 2^nd^ Trimester GWG/Week | 1.20 (0.74, 1.97) | 0.46 | 1.21 (0.72, 2.02) | 0.48 | 1.46 (0.89, 2.29) | 0.14 | 1.47 (0.87, 2.49) | 0.15 |
| 3^rd^ Trimester GWG/Week | **0.30 ± 0.11** | **<0.01** | **0.22 ± 0.11** | **0.05** | **0.23 ± 0.11** | **0.04** | 0.16 ± 0.11 | 0.16 |
| Excess 3^rd^ Trimester GWG/Week | **1.70 (1.01, 2.85)** | **0.04** | 1.50 (0.88, 2.55) | 0.14 | 1.23 (0.75, 2.02) | 0.41 | 1.06 (0.63, 1.79) | 0.83 |
| *Models adjusted for treatment assignment and baseline value of the activity variable  **Models adjusted for treatment assignment, baseline value of the activity variable, maternal age, race/ethnicity, parity, baseline BMI category  Odds ratios are per 1 SD increase in the activity/inactivity variable. | | | | | | | | |
