# Supplementary material for: LIFE-Moms: effects of multicomponent lifestyle randomized control trial on physical activity during pregnancy in women with overweight and obesity
Source: Int J Behav Nutr Phys Act. 2025 Sep 30;22:119. doi: 10.1186/s12966-025-01805-9 (PMC12486678; doi:10.1186/s12966-025-01805-9)
Supplement: Supplementary file 4 — Supplementary Material 4. [file 12966_2025_1805_MOESM4_ESM.docx]

| **Supplementary Table 4.** Relationship of gestational weight gain with sustaining/improving and worsening activity levels or activity levels in the bottom and top tertiles of change in activity levels across gestation. | | | | | | | | | | | | | | | |
| --- | --- | --- | --- | --- | --- | --- | --- | --- | --- | --- | --- | --- | --- | --- | --- |
|  | ***Sustained/Improved vs. Worsened Analysis*** | | | | | | |  | ***Bottom vs. Top Tertile Analysis^#^*** | | | | | | |
|  | ***Worsened***  ***(N=355)*** | | ***Sustained/ Improved (N=167)*** | |  | ***Unadjusted p-value**** | ***Adjusted p-value***** |  | ***Bottom***  ***(N=173)*** | | ***Top***  ***(N=171)*** | |  | ***Unadjusted p-value*** | ***Adjusted p-value**** |
|  | ***Mean ± SD or %*** | | ***Mean ± SD or %*** | |  |  |  |  | ***Mean ± SD or %*** | | ***Mean ± SD or %*** | |  |  |  |
| ***Total MVPA*** |  |  |  |  |  |  |  |  |  | |  | |  |  |  |
| *Gestational Weight Gain (kg)* | 8.85 | ± 5.23 | 8.95 | ± 4.76 |  | 0.808 | 0.966 |  | 8.86 | ± 5.17 | 9.03 | ± 4.95 |  | 0.716 | 0.983 |
| *Gestational Weight Gain/week (kg/wk)* | 0.39 | ± 0.23 | 0.39 | ± 0.21 |  | 0.872 | 0.921 |  | 0.39 | ± 0.23 | 0.40 | ± 0.21 |  | 0.722 | 0.949 |
| *Excess GWG per Week* | 65.9% | | 71.3% | |  | 0.201 | 0.207 |  | 65.9% | | 71.3% | |  | 0.251 | 0.230 |
| *2^nd^ Trimester GWG per Week (kg/wk)* | 0.38 | ± 0.26 | 0.38 | ± 0.23 |  | 0.910 | 0.858 |  | 0.38 | ± 0.25 | 0.39 | ± 0.25 |  | 0.753 | 0.748 |
| *Excess 2^nd^ Trimester GWG per Week* | 65.2% | | 64.9% | |  | 0.879 | 0.983 |  | 65.8% | | 65.2% | |  | 0.869 | 0.998 |
| *3^rd^ Trimester GWG per Week (kg/wk)* | 0.41 | ± 0.28 | 0.44 | ± 0.25 |  | 0.300 | 0.391 |  | 0.41 | ± 0.27 | 0.44 | ± 0.24 |  | 0.349 | 0.582 |
| *Excess 3^rd^ Trimester GWG per Week* | 66.5% | | 68.5% | |  | 0.693 | 0.674 |  | 71.4% | | 68.7% | |  | 0.554 | 0.458 |
| ***MVPA in bouts ≥ 1 minute*** |  |  |  |  |  |  |  |  |  |  |  |  |  |  |  |
| *Gestational Weight Gain (kg)* | 8.82 | ± 5.12 | 9.03 | ± 4.98 |  | 0.478 | 0.521 |  | 8.76 | ± 4.79 | 9.04 | ± 4.96 |  | 0.560 | 0.517 |
| *Gestational Weight Gain/week (kg/wk)* | 0.39 | ± 0.22 | 0.40 | ± 0.22 |  | 0.440 | 0.462 |  | 0.39 | ± 0.21 | 0.40 | ± 0.21 |  | 0.591 | 0.531 |
| *Excess GWG per Week* | 66.1% | | 71.2% | |  | 0.164 | 0.158 |  | 67.6% | | 71.1% | |  | 0.419 | 0.391 |
| *2^nd^ Trimester GWG per Week (kg/wk)* | 0.38 | ± 0.26 | 0.38 | ± 0.22 |  | 0.840 | 0.743 |  | 0.38 | ± 0.24 | 0.37 | ± 0.23 |  | 0.936 | 0.889 |
| *Excess 2^nd^ Trimester GWG per Week* | 64.2% | | 67.1% | |  | 0.377 | 0.260 |  | 69.1% | | 65.8% | |  | 0.608 | 0.809 |
| *3^rd^ Trimester GWG per Week (kg/wk)* | 0.41 | ± 0.26 | 0.43 | ± 0.28 |  | 0.327 | 0.374 |  | 0.40 | ± 0.25 | 0.43 | ± 0.28 |  | 0.298 | 0.291 |
| *Excess 3^rd^ Trimester GWG per Week* | 66.1% | | 69.6% | |  | 0.391 | 0.423 |  | 68.2% | | 69.4% | |  | 0.815 | 0.673 |
| ***Inactive time*** |  |  |  |  |  |  |  |  |  |  |  |  |  |  |  |
| *Gestational Weight Gain (kg)* | 8.55 | ± 5.18 | 9.47 | ± 4.86 |  | 0.056 | 0.187 |  | 9.60 | ± 4.66 | 8.40 | ± 4.70 |  | **0.015** | 0.057 |
| *Gestational Weight Gain/week (kg/wk)* | 0.38 | ± 0.23 | 0.42 | ± 0.21 |  | 0.054 | 0.155 |  | 0.42 | ± 0.21 | 0.37 | ± 0.21 |  | **0.021** | 0.059 |
| *Excess GWG per Week* | **63.6%** | | **74.7%** | |  | **0.011** | **0.018** |  | **75.9%** | | **65.1%** | |  | **0.026** | **0.044** |
| *2^nd^ Trimester GWG per Week (kg/wk)* | 0.37 | ± 0.25 | 0.40 | ± 0.26 |  | 0.173 | 0.319 |  | 0.41 | ± 0.25 | 0.37 | ± 0.24 |  | 0.095 | 0.171 |
| *Excess 2^nd^ Trimester GWG per Week* | 62.7% | | 69.4% | |  | 0.172 | 0.248 |  | 71.3% | | 62.5% | |  | 0.068 | 0.111 |
| *3^rd^ Trimester GWG per Week (kg/wk)* | 0.41 | ± 0.28 | 0.44 | ± 0.25 |  | 0.249 | 0.667 |  | 0.44 | ± 0.24 | 0.40 | ± 0.26 |  | 0.147 | 0.466 |
| *Excess 3^rd^ Trimester GWG per Week* | 66.7% | | 67.8% | |  | 0.837 | 0.795 |  | 69.0% | | 70.7% | |  | 0.766 | 0.460 |
| Summary variables presented as means ± SD or %. All models adjusted for the treatment assignment and included a random effect for site (protocol).  ^#^ Tertile ranges for each group are: Total MVPA Tertile 1 (Bottom): -106.4 minutes to -16.4 minutes, Tertile 3 (Top): -0.4 minutes to 66.7 minutes; MVPA in bouts ≥ 1 minute Tertile 1: -32.2 minutes to -6.1 minutes, Tertile 3: -0.5 minutes to 14.2 minutes; Inactive time Tertile 1: -181.0 minutes to -6.6 minutes, Tertile 3: 46.5 minutes to 251.0 minutes.  * Analysis adjusted for maternal age, race/ethnicity, parity, and baseline BMI category | | | | | | | | | | | | | | | |
