# Supplementary material for: LIFE-Moms: effects of multicomponent lifestyle randomized control trial on physical activity during pregnancy in women with overweight and obesity
Source: Int J Behav Nutr Phys Act. 2025 Sep 30;22:119. doi: 10.1186/s12966-025-01805-9 (PMC12486678; doi:10.1186/s12966-025-01805-9)
Supplement: Supplementary file 5 — Supplementary Material 5. [file 12966_2025_1805_MOESM5_ESM.docx]

| **Supplementary Table 5.** Relationship between activity level difference (end of pregnancy- baseline) and maternal and neonatal outcomes. | | | | | | | | | | | |
| --- | --- | --- | --- | --- | --- | --- | --- | --- | --- | --- | --- |
|  | | | *Change in Awake ENMO* | | | | | *Change in Total MVPA* | | | |
|  |  |  | *Unadjusted Model*** | *p-value* | | *Adjusted Model**** | *p-value* | *Unadjusted Model*** | *p-value* | *Adjusted Model**** | *p-value* |
| ***Pregnancy Complications*** | | |  |  | |  |  |  |  |  |  |
| Gestational Diabetes | | | 0.85 (0.62, 1.18) | 0.335 | | 0.87 (0.62, 1.22) | 0.406 | 0.77 (0.55,1.08) | 0. 122 | 0.77 (0.54, 1.10) | 0. 155 |
| Gestational Hypertension/Preeclampsia | | | 0.93 (0.68, 1.27) | 0.634 | | 0.91 (0.66, 1.25) | 0.551 | 0.92 (0.67, 1.27) | 0. 623 | 0.89 (0.64, 1.24) | 0.505 |
| Preeclampsia | | | 0.92 (0.57, 1.47) | 0. 721 | | 0.82 (0.50, 1.36) | 0. 450 | 0.92 (0.56, 1.50) | 0. 729 | 0.83 (0.49, 1.41) | 0. 486 |
| ***Maternal Biomarkers*** | | |  |  | |  |  |  |  |  |  |
| Glucose | | | -0.06 ± 0.05 | 0.213 | | -0.05 ± 0.05 | 0.322 | -0.03 ± 0.05 | 0.489 | -0.02 ± 0.05 | 0.720 |
| Insulin* | | | -0.06 ± 0.04 | 0.131 | | -0.06 ± 0.04 | 0.105 | -0.06 ± 0.04 | 0. 113 | -0.07 ± 0.04 | 0.094 |
| HOMA-IR* | | | -0.06 ± 0.04 | 0.134 | | -0.06 ± 0.04 | 0.114 | -0.06 ± 0.04 | 0.142 | -0.06 ± 0.04 | 0.130 |
| C-Peptide* | | | -0.02 ± 0.03 | 0. 604 | | -0.03 ± 0.03 | 0. 388 | -0.01 ± 0.03 | 0. 657 | -0.03 ± 0.03 | 0.426 |
| Total Cholesterol (mg/dL) | | | 0.08 ± 0.03 | **0.019** | | 0.06 ± 0.03 | 0. 075 | 0.09 ± 0.03 | **0.010** | 0.07 ± 0.03 | **0.030** |
| HDL-C (mg/dL) | | | 0.04 ± 0.04 | 0.250 | | 0.05 ± 0.04 | 0.176 | 0.04 ± 0.04 | 0.240 | 0.05 ± 0.04 | 0.195 |
| LDL-C (mg/dL) | | | 0.06 ± 0.04 | 0.099 | | 0.04 ± 0.04 | 0.246 | 0.07 ± 0.04 | 0.056 | 0.06 ± 0.04 | 0.107 |
| Triglycerides (mg/dL)* | | | 0.04 ± 0.03 | 0.162 | | 0.02 ± 0.03 | 0.430 | 0.04 ± 0.03 | 0.167 | 0.02 ± 0.03 | 0.351 |
| HMW Adiponectin (UNITS)* | | | 0.06 ± 0.03 | **0.039** | | 0.06 ± 0.03 | 0. 059 | 0.03 ± 0.03 | 0.339 | 0.03 ± 0.03 | 0.409 |
| TNF-α (UNITS) | | | -0.04 ± 0.04 | 0.292 | | -0.03 ± 0.04 | 0.415 | -0.03 ± 0.04 | 0.379 | -0.02 ± 0.04 | 0.557 |
| IL-6 (UNITS) | | | 0.00 ± 0.01 | 0.575 | | 0.00 ± 0.01 | 0.730 | 0.00 ± 0.01 | 0.714 | 0.00 ± 0.01 | 0.883 |
| Leptin (UNITS)* | | | -0.04 ± 0.04 | 0.336 | | -0.06 ± 0.04 | 0. 127 | 0.00 ± 0.04 | 0. 926 | -0.03 ± 0.04 | 0.504 |
| Maternal Biomarker Z-Score ^#^ | | | -0.10 ± 0.04 | **0.016** | | -0.11 ± 0.04 | **0.013** | -0.07 ± 0.04 | 0.103 | -0.08 ± 0.04 | 0.086 |
| Maternal Biomarker Z-Score^##^ | | | -0.05 ± 0.04 | 0.139 | | -0.06 ± 0.04 | 0.097 | -0.03 ± 0.04 | 0.400 | -0.03 ± 0.04 | 0.354 |
| ***Neonatal Outcomes*** | | |  |  | |  |  |  |  |  |  |
| Birth Weight (g) | | | -0.02 ± 0.05 | 0.720 | | -0.01 ± 0.05 | 0.768 | -0.03 ± 0.05 | 0.529 | -0.03 ± 0.05 | 0.559 |
| Head Circumference (cm) | | | -0.06 ± 0.05 | 0.230 | | -0.07 ± 0.05 | 0.156 | -0.08 ± 0.05 | 0.130 | -0.09 ± 0.05 | 0.079 |
| Triceps Skinfold (cm) | | | -0.06 ± 0.05 | 0.337 | | -0.03 ± 0.05 | 0.536 | -0.08 ± 0.06 | 0.129 | -0.07 ± 0.06 | 0.218 |
| Subscapular Skinfold (cm) | | | -0.06 ± 0.05 | 0.299 | | -0.03 ± 0.05 | 0.532 | -0.08 ± 0.05 | 0.139 | -0.06 ± 0.05 | 0.297 |
| Iliac Crest Skinfold (cm) | | | -0.05 ± 0.05 | 0.345 | | -0.04 ± 0.05 | 0.452 | -0.04 ± 0.05 | 0.495 | -0.03 ± 0.05 | 0.586 |
| Thigh Skinfold (cm) | | | -0.00 ± 0.05 | 0.957 | | 0.02 ± 0.05 | 0.659 | -0.03 ± 0.05 | 0.571 | -0.01 ± 0.05 | 0.902 |
| Birth Weight for Length Z-score | | | -0.04 ± 0.05 | 0.432 | | -0.02 ± 0.05 | 0.646 | -0.02 ± 0.05 | 0.631 | -0.00 ± 0.05 | 0.961 |
| Estimated Percent Fat Mass (%) | | | -0.01 ± 0.05 | 0.835 | | -0.00 ± 0.05 | 0.977 | -0.01 ± 0.05 | 0.809 | -0.00 ± 0.05 | 0.981 |
| Small for Gestational Age (< 10^th^ %) | | | 0.99 (0.68, 1.43) | 0.944 | | 0.89 (0.60, 1.32) | 0.546 | 1.00 (0.68, 1.46) | 0.999 | 0.89 (0.59, 1.35) | 0.573 |
| Large for Gestational Age (> 90^th^ %) | | | 0.77 (0.56, 1.08) | 0.134 | | 0.83 (0.58, 1.16) | 0.272 | 0.77 (0.54, 1.08) | 0.130 | 0.83 (0.59, 1.19) | 0.310 |
|  | | | *Change in MVPA Time in Bouts ≥ 1 Minute^†^* | | | | | *Change in Inactive Time** | | | |
|  | *Unadjusted Model*** | | | *p-value* | *Adjusted Model**** | *p-value* | *Unadjusted Model*** | *p-value* | *Adjusted Model**** | *p-value* |  |
| ***Pregnancy Complications*** |  | | |  |  |  |  |  |  |  |  |
| Gestational Diabetes | 0.72 (0.49, 1.07) | | | 0.100 | 0.76 (0.51, 1.14) | 0.179 | 1.40 (0.78, 2.48) | 0.256 | 1.31 (0.72, 2.39) | 0.383 |  |
| Gestational Hypertension/Preeclampsia | 1.08 (0.76, 1.53) | | | 0.662 | 1.05 (0.73, 1.51) | 0.791 | 0.81 (0.46, 1.45) | 0.480 | 0.88 (0.48, 1.60) | 0.665 |  |
| Preeclampsia | 0.91 (0.52, 1.59) | | | 0.744 | 0.81 (0.44, 1.49) | 0.498 | 1.31 (0.55, 3.07) | 0.542 | 1.72 (0.67, 4.37) | 0.258 |  |
| ***Maternal Biomarkers*** |  | | |  |  |  |  |  |  |  |  |
| Glucose | -0.08 ± 0.05 | | | 0.165 | -0.05 ± 0.05 | 0.362 | 0.16 ± 0.09 | 0.078 | 0.16 ± 0.09 | 0.079 |  |
| Insulin* | -0.04 ± 0.04 | | | 0.375 | -0.04 ± 0.04 | 0.342 | 0.11 ± 0.07 | 0.123 | 0.12 ± 0.07 | 0.082 |  |
| HOMA-IR* | -0.04 ± 0.04 | | | 0.340 | -0.04 ± 0.05 | 0.339 | 0.12 ± 0.07 | 0.094 | 0.14 ± 0.07 | 0.062 |  |
| C-Peptide* | 0.01 ± 0.04 | | | 0.880 | -0.00 ± 0.04 | 0.919 | 0.06 ± 0.06 | 0.346 | 0.08 ± 0.06 | 0.146 |  |
| Total Cholesterol (mg/dL) | 0.10 ± 0.04 | | | **0.007** | 0.09 ± 0.04 | **0.019** | -0.15 ± 0.06 | **0.016** | -0.10 ± 0.06 | 0.087 |  |
| HDL-C (mg/dL) | 0.09 ± 0.04 | | | **0.038** | 0.09 ± 0.04 | **0.027** | -0.06 ± 0.06 | 0.412 | -0.08 ± 0.07 | 0.268 |  |
| LDL-C (mg/dL) | 0.06 ± 0.04 | | | 0.109 | 0.06 ± 0.04 | 0.186 | -0.12 ± 0.07 | 0.082 | -0.08 ± 0.07 | 0.250 |  |
| Triglycerides (mg/dL)* | 0.04 ± 0.03 | | | 0.236 | 0.03 ± 0.03 | 0.395 | -0.08 ± 0.05 | 0.107 | -0.03 ± 0.05 | 0.464 |  |
| HMW Adiponectin (UNITS)* | 0.02 ± 0.03 | | | 0.473 | 0.02 ± 0.03 | 0.532 | -0.09 ± 0.05 | 0.089 | -0.07 ± 0.06 | 0.199 |  |
| TNF-α (UNITS) | -0.04 ± 0.04 | | | 0.289 | -0.03 ± 0.04 | 0.491 | 0.03 ± 0.07 | 0.685 | 0.02 ± 0.07 | 0.797 |  |
| IL-6 (UNITS) | 0.00 ± 0.01 | | | 0.980 | -0.00 ± 0.01 | 0.762 | 0.01 ± 0.01 | 0.410 | 0.01 ± 0.01 | 0.253 |  |
| Leptin (UNITS)* | 0.02 ± 0.05 | | | 0.682 | -0.02 ± 0.05 | 0.612 | 0.11 ± 0.08 | 0.170 | 0.14 ± 0.08 | 0.061 |  |
| Maternal Biomarker Z-Score ^#^ | -0.06 ± 0.05 | | | 0.199 | -0.07 ± 0.05 | 0.170 | 0.22 ± 0.08 | **0.004** | 0.23 ± 0.08 | **0.003** |  |
| Maternal Biomarker Z-Score^##^ | -0.03 ± 0.04 | | | 0.521 | -0.03 ± 0.04 | 0.457 | 0.12 ± 0.07 | 0.075 | 0.15 ± 0.07 | **0.028** |  |
| ***Neonatal Outcomes*** |  | | |  |  |  |  |  |  |  |  |
| Birth Weight (g) | 0.02 ± 0.05 | | | 0.776 | -0.02 ± 0.06 | 0.788 | -0.07 ± 0.09 | 0.486 | -0.03 ± 0.09 | 0.774 |  |
| Head Circumference (cm) | -0.04 ±0.06 | | | 0.473 | -0.05 ± 0.06 | 0.366 | 0.03 ± 0.09 | 0.722 | 0.08 ± 0.09 | 0.357 |  |
| Triceps Skinfold (cm) | -0.07 ± 0.06 | | | 0.259 | -0.06 ± 0.06 | 0.342 | 0.01 ± 0.10 | 0.898 | -0.02 ± 0.10 | 0.861 |  |
| Subscapular Skinfold (cm) | -0.07 ± 0.06 | | | 0.255 | -0.05 ± 0.06 | 0.448 | 0.02 ± 0.10 | 0.842 | -0.01 ± 0.10 | 0.910 |  |
| Iliac Crest Skinfold (cm) | -0.05 ± 0.06 | | | 0.444 | -0.04 ± 0.06 | 0.456 | 0.00 ± 0.10 | 0.999 | -0.02 ± 0.10 | 0.835 |  |
| Thigh Skinfold (cm) | -0.05 ± 0.06 | | | 0.371 | -0.04 ± 0.06 | 0.559 | -0.06 ± 0.10 | 0.581 | -0.10 ± 0.10 | 0.309 |  |
| Birth Weight for Length Z-score | -0.15 ± 0.05 | | | **0.007** | -0.12 ± 0.05 | **0.032** | 0.09 ± 0.09 | 0.295 | 0.08 ± 0.09 | 0.384 |  |
| Estimated Percent Fat Mass (%) | -0.01 ± 0.06 | | | 0.819 | -0.00 ± 0.06 | 0.953 | -0.05 ± 0.10 | 0.587 | -0.04 ± 0.10 | 0.690 |  |
| Small for Gestational Age (< 10^th^ %) | 1.12 (0.75, 1.66) | | | 0.586 | 1.01 (0.66, 1.56) | 0.958 | 0.96 (0.48, 1.94) | 0.915 | 1.09 (0.52, 2.26) | 0.822 |  |
| Large for Gestational Age (> 90^th^ %) | 0.84 (0.58, 1.22) | | | 0.369 | 0.95 (0.65, 1.38) | 0.779 | 1.05 (0.58, 1.92) | 0.873 | 1.01 (0.54, 1.89) | 0.971 |  |
| Presented as β ± SE or OR (95% CI). Odds ratios are per 1 SD increase in the activity/inactivity variable.  *Log transform used for modeling to obtain approximate normality  **Models only adjusted for treatment assignment, the baseline value for the independent variable (activity or inactivity measure), and, for the biomarkers, the baseline value for the dependent variable  ***Models adjusted for treatment assignment, the baseline value for the independent variable (activity or inactivity measure), maternal age, race/ethnicity, parity, baseline BMI category, and, for the biomarkers, the baseline value of the dependent variable  # Z-score constructed from the standardized sum of the following selected biomarkers: glucose, insulin, leptin, IL-6, TNF-α, HOMA-IR, and high molecular weight adiponectin (negative)  #Z-score constructed from the standardized sum of all biomarkers: insulin, glucose, C-peptide, leptin, total cholesterol, LDL cholesterol, triglycerides, IL-6, TNF-α, and high molecular weight adiponectin (negative) and HDL cholesterol (negative) | | | | | | | | | | |  |
