# Supplementary figures and images for: LIFE-Moms: effects of multicomponent lifestyle randomized control trial on physical activity during pregnancy in women with overweight and obesity
Source: Int J Behav Nutr Phys Act. 2025 Sep 30;22:119. doi: 10.1186/s12966-025-01805-9 (PMC12486678; doi:10.1186/s12966-025-01805-9)

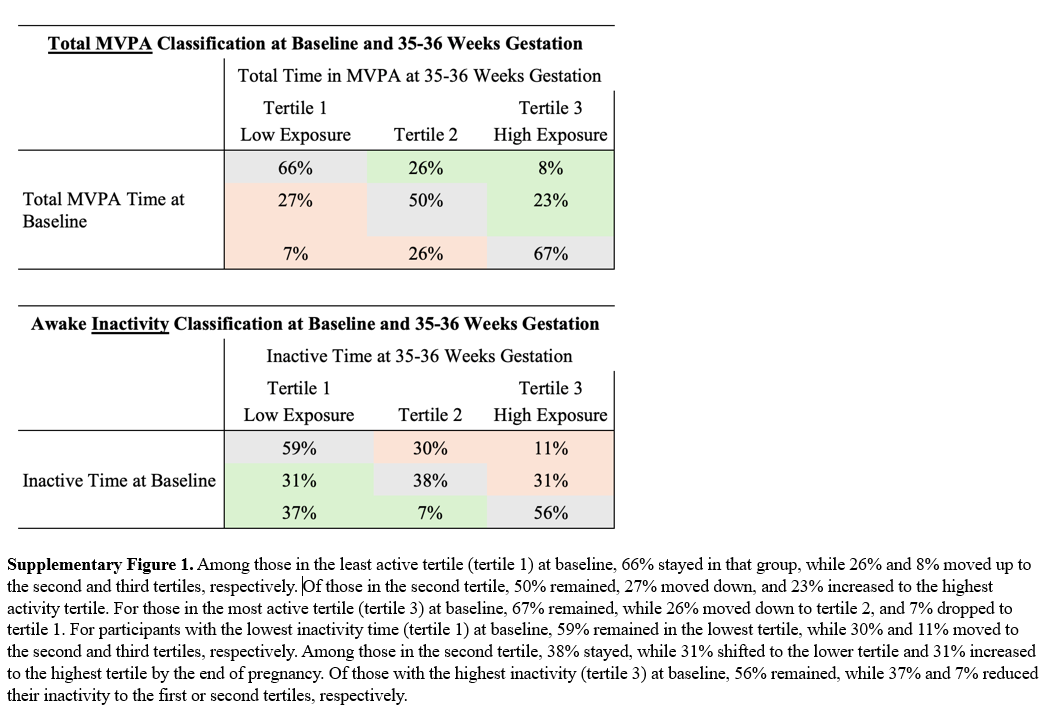

Supplement: Supplementary file 6 — Supplementary Material 6. [file 12966_2025_1805_MOESM6_ESM.png]
